# Course of Mental Health in Refugees—A One Year Panel Survey

**DOI:** 10.3389/fpsyt.2018.00352

**Published:** 2018-08-03

**Authors:** Elisa Kaltenbach, Maggie Schauer, Katharin Hermenau, Thomas Elbert, Inga Schalinski

**Affiliations:** Clinical Psychology and Clinical Neuropsychology, Universität Konstanz, Konstanz, Germany

**Keywords:** refugee, mental disorders, longitudinal study, PTSD, depression, traumatic experiences, postmigrational stressor, Germany

## Abstract

**Background:** Cross-sectional studies indicate that a substantial proportion of refugees have psychiatric disorders. However, longitudinal studies on the course of psychiatric symptoms and on influencing factors are scarce. The current study investigates the development of symptoms in an untreated refugee sample in Germany and seeks to identify potential predictors.

**Methods:** Over the course of 1 year, 57 refugees participated in monthly assisted self-reports on the phone assessing emotional distress. At the same time, semi-annual, semi-structured clinical interviews focusing on posttraumatic stress disorder (PTSD) and depression were conducted. The overall dropout rate for the year was 23% for the assisted self-reports and 33% for the clinical interviews.

**Results:** Symptoms did not systematically change over the course of the year. On the individual level, a reliable change in PTSD symptoms was observed in 13% who showed improvement and 24% who showed worsening symptoms. Figures for depression symptoms were 24 and 16% respectively. A higher number of traumatic experiences was related to a greater intensity of PTSD symptoms. In addition, postmigrational stressors were associated with a worsening of PTSD symptoms over the course of the year. Emotional distress was associated with current negative life events, unemployment, and frequent visits to physicians.

**Conclusions:** There is on average no improvement or worsening of symptoms over the period of 1 year. However, individual courses vary, and thus show the importance of risk factors. Accordingly, the identification of risk factors such as trauma load and postmigrational stressors can be useful to determine the need of further monitoring and to provide appropriate interventions when necessary.

## Introduction

Increasing numbers of refugees worldwide have been recorded in the last years (1). In a careful meta-analysis of studies examining rates of psychiatric disorders in samples of refugees relocated to high-income western countries, Fazel et al. (2) emphasized the heterogeneity of the samples and of the findings. From these data, Miller et al. (3) concluded that about a third of the refugees in well-to-do countries present with PTSD. However, these authors also argued that a general, standard rate of PTSD in refugees worldwide may not be meaningful, as the likelihood of developing PTSD depends largely on the cumulative exposure to traumatic stressors (4, 5) as does its spontaneous remission (6). Recent reviews and studies with current refugee groups in Germany and other European countries showed similar prevalence rates (7–12). Research on the long-term mental health problems of refugees also showed a high variability and high prevalences, with depression rates ranging between 2 and 80% and PTSD rates between 4 and 86% (13). In certain conditions, PTSD or depression can affect half of the refugees even one to two decades after resettlement (14–16).

While most research in this area is conducted in the form of cross-sectional designs, longitudinal studies are essential for designing healthcare. Findings of longitudinal studies in refugee populations vary immensely—results range from improving to unchanging to aggravating mental health symptoms over time. Several studies showed improvements in the refugees' mental health symptoms, for example, a decline in the PTSD and depression rate of 55% within 3 years (17–19). On the other hand, a few studies found an overall increase in PTSD symptoms over time—for example, from 37 to 80% within 1.5 years (20, 21). Other studies reported no overall change in mental health symptoms, but highlighted the high variation in symptoms within persons over time (22, 23). For example, Lamkaddem et al. (24) found no change in PTSD prevalence after 7 years; however, half of the refugees being diagnosed with PTSD at the 7 years follow-up were new cases.

Refugees frequently experienced adversities such as domestic violence and poverty during their upbringing (1, 25, 26). Furthermore, they often faced organized violence in their home country, such as war and torture, and criminal violence during their flight (1). All of these factors are known to contribute to mental ill-health (27–29). Additionally to the exposure to traumatic stressors, it has been argued that postmigrational stressors (PMS)—especially social and interpersonal factors, but also factors connected to the asylum procedure and socioeconomical situation—may play a major role in the development and course of PTSD, depression, and anxiety disorders in refugees (13, 30–32).

To our knowledge, there are no longitudinal studies on the naturalistic course of refugees' mental health across extended periods. Thus, we aimed to examine the course of mental health symptoms in an untreated refugee sample living in Germany. We combined semi-structured clinical interviews assessing PTSD and depression, conducted semi-annual, with monthly assisted self-ratings on emotional distress (screening for symptoms of PTSD, depression, and anxiety). Furthermore, we intended to reveal potential predictor variables influencing the course of these symptoms. Therefore, we examined the association of traumatic experiences and PMS with the changes in mental health. As research on daily stressors and their effect on mental health is scarce, we moreover analyzed the effect of daily stressors on emotional distress. We also aimed to detect further variables associated with mental health problems such as higher numbers of medical visits.

## Method

### Study design

To closely monitor the course of mental health symptoms in refugees, the current study consisted of two complementary parts:

(1) Monthly assisted self-report (t1–t12): Monthly telephone interviews were conducted over the course of 1 year and started 1 month after the first clinical interview (t0, see below). Trained native speakers conducted an interview with the participating refugees using a self-report instrument about emotional distress and life changes over the past month. The duration was around 15 min.

(2) Semi-annual clinical interviews (t0, t6, t12): Expert psychologists performed semi-structured clinical interviews at baseline (t0), after 6 months (t6), and after 12 months (t12). The initial interview (t0) was conducted within the scope of other studies performed at the Center of Excellence for Psychotraumatology (CEP) and took around 2–4 h to complete, often split in two sessions. The interviews at t6 and t12 took around 1 to 2 h to complete. All interviews included questionnaires about posttraumatic stress disorder (PTSD) and depression. Additionally, traumatic experiences were assessed at t0, and postmigrational stressors at t12.

**Figure 1:**
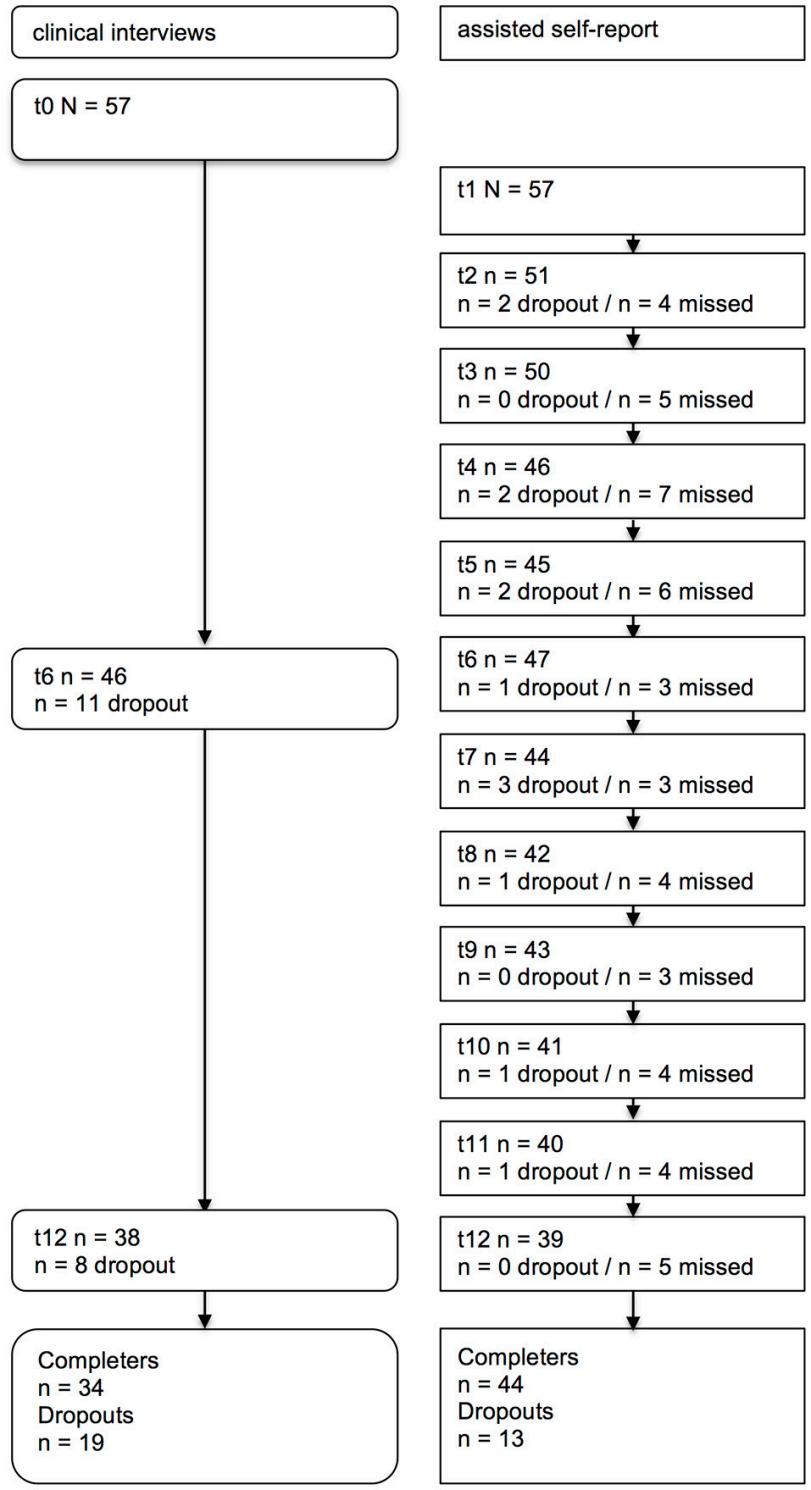
Flow of participants through the study. Clinical interviews = half-yearly semi-structured clinical interviews, assisted self-report = monthly assisted self-report on the telephone, dropout = participants dropping out of the study at the specific time point, missed = participants who missed an interview at the specific time point but who continued their participation.

The study design and the flow of participants is depicted in Figure 1. Dropout was defined as the last point in time a person participated in the study. Missed was defined as one absent time point of a person who is still participating in the study. Completers for the assisted self-report were defined as those who participated in at least 8 interviews or in all 3 time points for the clinical interview, respectively. The dropout rate for the assisted self-report was 23% (*n* = 13). The reasons were deportation to the home countries (*n* = 5), the wish to end the participation (*n* = 4), and unavailability on the phone (*n* = 4). The dropout rate for the clinical interviews was somewhat higher (33%; *n* = 19) due to the relocation of some participants to distant regions (*n* = 6). Characteristics between study completers and dropouts were comparable in most aspects; however, completers had a shorter duration of stay in Germany and a higher number of traumatic event types compared to dropouts (for further information, see Supplementary Table 1).

### Procedures

Most refugees of the present study were actively recruited by research assistants in asylum accommodations in and surrounding a medium-sized city in southern Germany. Some were referred to the CEP by social workers, volunteers, or lawyers because they were conspicuous in their behavior. After the initial interview (t0), the further procedure was discussed with the participants.

Participants with mental health problems were thoroughly informed about their symptoms and if wished, they either were offered therapy at the CEP or they were referred to other institutions. Only refugees who did not receive psychological treatment were included in the study. Some of the refugees showed clinically-relevant symptoms but either (a) were put on our waiting list because of limited capacities of the CEP and when offered a therapy after some months, they did not start a therapy (because they did not fulfill the PTSD criteria anymore, moved away, or had no time because of their job or family issues), or (b) did not wish a treatment at that point in time. At the end of the study some of the participants wished and accordingly received treatment. Refugees who accepted psychological treatment at the CEP were also monitored, but this investigation is still ongoing. No more than 2 persons of the same family were included, this was the case for 5 dyads. Written informed consent was given by the participants, and in case of minors, additionally by the legal guardian. Participants received an information sheet with relevant information on the study. Monetary compensation (€40) was given for their contribution. In case of arising problems, participants were able to contact the investigators and, if necessary, an additional appointment was arranged. The Ethical Review Board of the University of Konstanz, Germany approved the study. Including the participants receiving psychological treatment, the project was registered at Clinical Trials (clinicaltrials.gov) with the registration number NCT02852616. The goal of the present investigation was not to evaluate treatment success, but to closely monitor changes in those who do not seek treatment.

### Setting

The study was conducted at the CEP between 2015 and 2017. Each participant was monitored for a period of 12 months. The CEP is a specialized research center for asylum seekers and refugees, which in particular studies the effects of traumatic stress on mental health and offers treatment to refugees with severe trauma-related suffering. The clinical interviews were conducted by clinical psychologists trained and experienced in the work with refugees and the diagnostic of mental health problems. Most interviews were conducted with the help of experienced interpreters who were also trained and supervised in this context. To ensure blindness in respect to the prior mental health status, we aimed to assign a different interviewer and interpreter for each clinical interview of one participant. However, the available interpreters for languages rarely requested were below three, resulting in a limited blindness of the interpreters for some participants.

The assisted self-report was conducted by native speakers who received a training especially designed for the purpose of this study (*n* = 23). The training included theoretical and practical lectures on mental health issues, how to conduct an assisted self-report as a telephone interview, how to handle possible upcoming problems, and supervised practicing of the assisted self-report. Supervision was provided regularly. Participants had the same interviewer for all assisted self-reports. However, due to the unavailability of five interviewers in the course of the study, changes in the interviewer were necessary for the according participants. The phone calls for the assisted self-reports were conducted at the CEP or the homes of the interviewers without the presence of other people. To create a confidential setting, respondents were asked to find a calm and undisturbed place. On average, telephone interviewers tried to contact the participant *M* = 2.9 (*SD* = 2.4, range 1–20) until the interview could be successfully completed. The assisted self-reports took on average *M* = 18.6 min (*SD* = 7.4, range 5–45).

### Participants

Fifty-seven refugees and asylum seekers participated in the study. Inclusion criteria were status as asylum seeker or refugee and age above 12 years. Exclusion criteria were continuous psychotherapy, presence of acute psychotic symptoms, or no access to a phone. Sociodemographic characteristics are depicted in Table 1.

**Table 1:** Socio-demographic characteristics.

**Characteristics**	**Total sample (*N* = 57)**
Female sex, No. (%)	34 (60)
Age, *M* (*SD*, range), years	30.3 (11.5, 12–56)
Adolescent, No. (%)	8 (14)
Education, *M* (*SD*, range), years	8.0 (4.1, 0–15)
Stay in Germany, *M* (*SD*, range), months	9.3 (6.6, 2–36)
Core family members in Germany, No. (%)	41 (72)
**COUNTRY OF ORIGIN, NO. (%)**	
Syria, No. (%)	22 (39)
Afghanistan, No. (%)	15 (26)
Iran, No. (%)	7 (12)
Balkan states, No. (%)	6 (11)
Iraq, No. (%)	4 (7)
West Africa, No. (%)	2 (4)
Russia, No. (%)	1 (2)
**ACCOMMODATION, NO. (%)**	
Emergency shelter	23 (40)
Standard refugee accommodation	22 (39)
Private accommodation	12 (21)
**ASYLUM STATUS, NO**. (%)	
First instance application	40 (70)
Rejection	9 (16)
Recognition	8 (14)

### Measures

#### Assisted self-reports

The assisted self-reports consisted of questions about daily life, changes in the past month and the Refugee Health Screener−13. Questionnaires were identical for all 12 interviews.

##### Daily life and changes in the past month

Five self-constructed questions about the participants' occupation, life events, medical visits, and changes in the accommodation and the asylum procedure were asked. If participants answered with yes, subsequent in-depth questions were asked (e.g., which kind of change occurred, what kind of occupation they had; see Supplementary Table 2 for the wording of the questions). For calculations, occupation was defined as a regularly attended work, apprenticeship, or school. Only a few people reported changes in their housing situation or asylum status; accordingly, no calculations with these factors could be conducted. New life events were split into positive and negative events. For visits to physicians, the number of visits to the general practicioner and other medical doctors (psychiatrists were excluded) were summarized. Visits to psychotherapists, psychiatrists, or inpatient health care were rarely reported and therefore not included in the calculations.

##### Refugee health screener−13

The Refugee Health Screener−13 [RHS-13; (33)] is a shortened 13-item version of the original RHS-15 (34). It is an efficient self-rating instrument which assesses emotional distress in refugees, thereby comprising symptoms of PTSD, depression, and anxiety. The 13-item version shows good psychometric properties in refugee samples in the US and in Germany, comparable to the characteristics of the RHS-15 (8, 9, 33). Cronbach's α varied between 0.89 and 0.95 for the 12 time points. The RHS-13 correlated significantly with the measures used for PTSD and depression symptoms at the according time points (PTSD: *r*_t01_ = 0.33 (*p* < 0.05); *r*_t06_ = 0.55 (*p* < 0.001); *r*_t12_ = 0.45 (*p* < 0.01); depression: *r*_t01_ = 0.43 (*p* < 0.001); *r*_t06_ = 0.63 (*p* < 0.001); *r*_t12_ = 0.52 (*p* < 0.001)).

#### Semi-structured clinical interviews

All the instruments used in the semi-structured clinical interviews are described below and are organized thematically.

##### Sociodemographic data

Sociodemographic information was asked in detail at t0 and included, inter alia, sex, age, education, country of origin, duration of stay in Germany, type of accommodation, and asylum status.

##### PTSD

Due to the change from DSM-IV to DSM-5, PTSD, and traumatic events were assessed with two differing instruments in t0. Thereby, we applied one PTSD instrument for each participant. For those participating at the beginning of the study, the PTSD Symptom Scale—Interview Version [PSS-I; (35)] for DSM-IV was used. It consists of 17 questions and is rated on a 4-point Likert scale. The PSS-I shows good psychometric properties (36, 37). For DSM-5, the Posttraumatic Stress Disorder Checklist-5 [PCL-5; (38)] was used. It consists of 20 questions and is rated on a 5-point Likert scale. Studies show a good validity and reliability for the PCL-5 (39, 40). Both PSS-I and PCL-5 were administered as a semi-structured clinical interview, including severity and frequency of the symptoms in equal parts. Each item which was rated with at least “moderately” was considered for the evaluation of the DSM-5 criteria. Additionally, we assessed the remaining criteria (duration of symptoms and functionality). In the 6- and 12-month follow-up interviews, the PCL-5 (DSM-5) was used consistently.

The PSS-I and PCL-5 have a different scaling (PSS-I: 0–3, PCL-5: 0–4), therefore the original scores needed to be transformed for calculations. As the majority of the data was assessed with the PCL-5, we transformed the PSS-I scale to make it comparable to the PCL-5 scale. Following Wortmann et al. (41) who have done the same transformation, the PSS-I scale was transformed by multiplying the original values with 4/3. The three items that were not assessed in the PSS-I were imputed using the individual mean of all measured items. The total sum score of the PCL-5 (range 0–80) and diagnosis according to DSM-5 are used in this study. Cronbach's α at t0 is 0.89, at t6 0.95, and at t12 0.90.

##### Depression

The Patient Health Questionnaire −9 [PHQ-9; (42)] was used at t0, t6, and t12. It assesses all 9 depression symptoms on a 4-point Likert scale. The PHQ-9 is a commonly used instrument and shows good psychometric properties (42, 43). Cronbach's α at t0, t6, and t12 is 0.87, 0.90, and 0.85. We used the symptom severity and diagnosis according to DSM-5 in this study. Depression diagnosis was met when at least 5 items were rated to be present “more than half the days” or “nearly every day” in the past 2 weeks, with at least one of those items being item 1 or 2 (depressed mood or loss of interest/ pleasure).

##### Traumatic events

Traumatic events were assessed in detail at t0 with two different instruments: The event checklist of the PSS-I (44) contains 12 different traumatic events types and was used in combination with the PSS-I. The Life Events Checklist [LEC-5; (45)] was used in combination with the PCL-5. It contains 17 categories of traumatic events and shows good psychometric properties (46). For each participant only one of the two questionnaires was assessed. For calculations, the overlapping 12 events which were experienced or witnessed by the participants were used. To also include all events named by the participants where the LEC-5 was used, namings in the additional 5 items were re-sorted to the most suitable of the 12 events. Calculations were conducted with the sum score of the 12 dichotomous items. The occurrence of new traumatic events was assessed in t6 and t12.

##### Postmigrational stressors

Postmigrational stressors (PMS) were assessed using the Post-Migration Living Difficulties Checklist [PMLD; (47)] at t12. The checklist was especially developed for refugees and has been used in diverse refugee populations (48). It consists of 24 potential PMS and is rated on a 5-point Likert scale for the last 12 months. Items can be grouped into three domains: Protection concerns (e.g., fear of being sent home, delays in processing applications, worries about family at home), access to health and welfare (e.g., poor access to emergency care, little governmental help), and stress related to resettlement conditions (e.g., not being able to find work, poverty, loneliness and boredom; (22)). To meet the particular circumstances of the refugee population in Germany, one item called “living situation” was added. No change in Cronbach's α = 0.83 could be detected by adding the item. For calculations the sum score ranging from 0 to 100 was used.

### Data analysis

The statistical program SPSS 24.0 supported the descriptive data analysis and the program R 3.3.2 was used for statistical calculations such as linear growth models, multiple imputation, and for graphical displays. To check if participants who dropped out of the study differed in their characteristics to those who stayed in the study, *t*-tests for independent samples, Mann-Whitney U tests, and LR χ^2^ tests were conducted.

#### Individual symptom changes

Meaningful symptom changes in the individuals were calculated with the reliable change index [RCI; (49)]. For the calculations, the test-retest reliability of *r* = 0.82 for the PCL-5 (50) and *r* = 0.84 for the PHQ-9 (51) was used. Further, the SD of the baseline assessment of the present sample was included. Significant improvement or worsening of symptoms was considered as statistically significant if the difference between t0 and t12 exceeded 1.96 (α = 0.05). Participants were divided in four groups: improvement, worsening, ongoing clinically relevant symptoms without a significant change (defined as fulfilling the diagnosis according to DSM-5 at least at one of the two included time points), and subclinical symptoms without a significant change.

#### Linear growth models

Linear growth models (LGM; also called mixed effects or multi-level models) offer an appealing approach to analyse longitudinal data by including within- and between-subject changes (52). In the present study, LGMs were used to assess potential changes in symptoms in the course of time as well as to identify possible predictors. Calculations were conducted with the nlme package in R (53).

We calculated LGMs for the response variables RHS-13, PCL-5, and PHQ-9. Fixed and random effect components were specified in the following manner:

(a) Fixed factors: Time, possible predictors, and their interaction were considered as fixed main effects. Analyzed predictor variables for RHS-13, PCL-5, and PHQ-9 were sociodemographic variables, traumatic events, and PMS. Additional predictor variables for the RHS-13 were occupation, positive and negative events, and medical visits. We only allowed two factors and their interaction per model to avoid overfitting. To control for the cumulated alpha error due to multiple testing, we applied the Benjamini-Hochberg procedure (54), resulting in a corrected *p*-value of α = 0.013 for the RHS-13 and α = 0.025 for the PCL-5. Because there were no close to significant results for the PHQ-9, no corrected *p*-valued could be calculated.

(b) Random factors: For each fixed effect model, random intercept and random slope model were compared. First, we added a random intercept which included the variable *subject*, thereby allowing each participant to have an individually different initial level of symptoms. In a second step, we formulated a random slope model which included the variables *subject* and *time*. Additionally to the random intercept, the random slope allows for individual changes in symptoms over time. To determine the model with the best fit, the random intercept and slope model were then compared using the likelihood ratio χ^2^ test. In case of a non-significant difference between the models, and following the law of parsimony, the random intercept model was chosen. Further, we included the autocorrelation structure AR(1) because symptoms of adjacent time points are likely to correlate to a higher degree (52). As an estimator, the restricted maximum likelihood estimator was used because it accounts for the uncertainty in the fixed effects and is therefore preferable in small samples (55).

The assumptions of LGMs were met (55). Following Maas and Hox (56), LGMs also show accurate results in small sample sizes comparable to the present sample. The inclusion of all available data (*N* = 57 participants) was allowed because LGMs can handle missing data that are missing completely at random (the missings are completely unrelated to the data) or missing at random (the missings are not related to the missing values itself but to other variables; 55). Visual inspection of the raw values led us to the conclusion that the data in the present study was found to be missing at random. To check the stability of the results, we compared the LGMs with all participants included vs. the LGMs excluding participants with less than 2/3 of the data. No differences could be detected; therefore, the results for the whole sample will be reported. To graphically illustrate the course of symptoms, we used spaghetti plots and exemplary individual courses of symptoms as described in Bolger and Laurenceau (52).

#### Missing values

Missing values are a difficulty often faced in multilevel data (57). The replacement of missings was done with multiple imputation (MI)—a commonly used and recommended method in estimating missing values in multilevel data (58). MI was applied with the help of the R package mice 2.30 (59). The assumption that the missing data can be classified as missing at random can be seen as fulfilled by visual inspection (60). Missing values in individual scores were imputed to calculate sum scores of response variables when less than 10% of the according questionnaire was missing. Additionally, missing time points were imputed for the graphical presentation of the exemplary individual courses of symptoms if <1/3 of the data was missing. This led to a reduced sample size of *n* = 44 for the RHS-13 and *n* = 34 for PCL-5 and PHQ-9. For the LGMs no MI was conducted. As the missing 3 items in the PSS-I don't fulfill the assumption of random missingness, we imputed these items with the individual mean.

## Results

### Traumatic experiences, postmigrational stressors, and mental health

Participants experienced between 1 and 12 different traumatic event types (*M* = 4.9, *SD* = 2.6, *n* = 57). Postmigrational stressors (PMS) were rated on average with *M* = 30.1 (*SD* = 14.9, range 4–75, *n* = 38). A PTSD was diagnosed for 32% (18 of 57 cases; PCL-5 score *M* = 15.4, *SD* = 12.6, range 0–48) at t0, for 27% (12 of 44; *M* = 18.6, *SD* = 16.6, range 0–60) at t6, and 24% (9 of 38; *M* = 16.7, *SD* = 12.5, range 0–51) of the participants at t12. Criteria for a major depression were fulfilled by 16% (9 of 57; PHQ score *M* = 8.2, *SD* = 6.1, range 0–27) at t0, by 27% (12 of 45; *M* = 9.5, *SD* = 7.0, range 0–25) at t6, and by 16% (6 of 38; *M* = 7.4, *SD* = 5.7, range 0–22) of the participants at t12.

### Course of mental health symptoms

**Figure 2:**
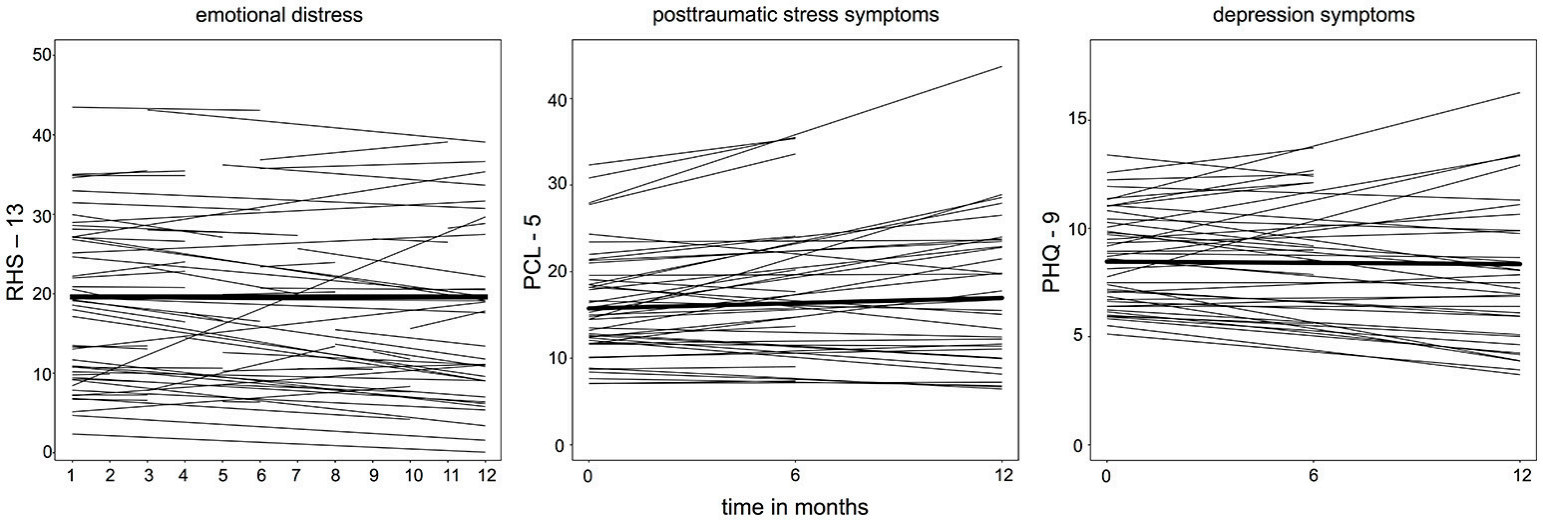
Spaghetti plots derived from the linear growth models (LGM) for emotional distress, PTSD, and depression. Bold lines represent the intercept and course of an average person. Each thin line represents the fitted regression line for one subject. For the 3 LGMs with the according response variables RHS-13, PCL-5, and PHQ-9, the fixed factor was time, and the random factors were subject and time (random slope).

**Figure 3:**
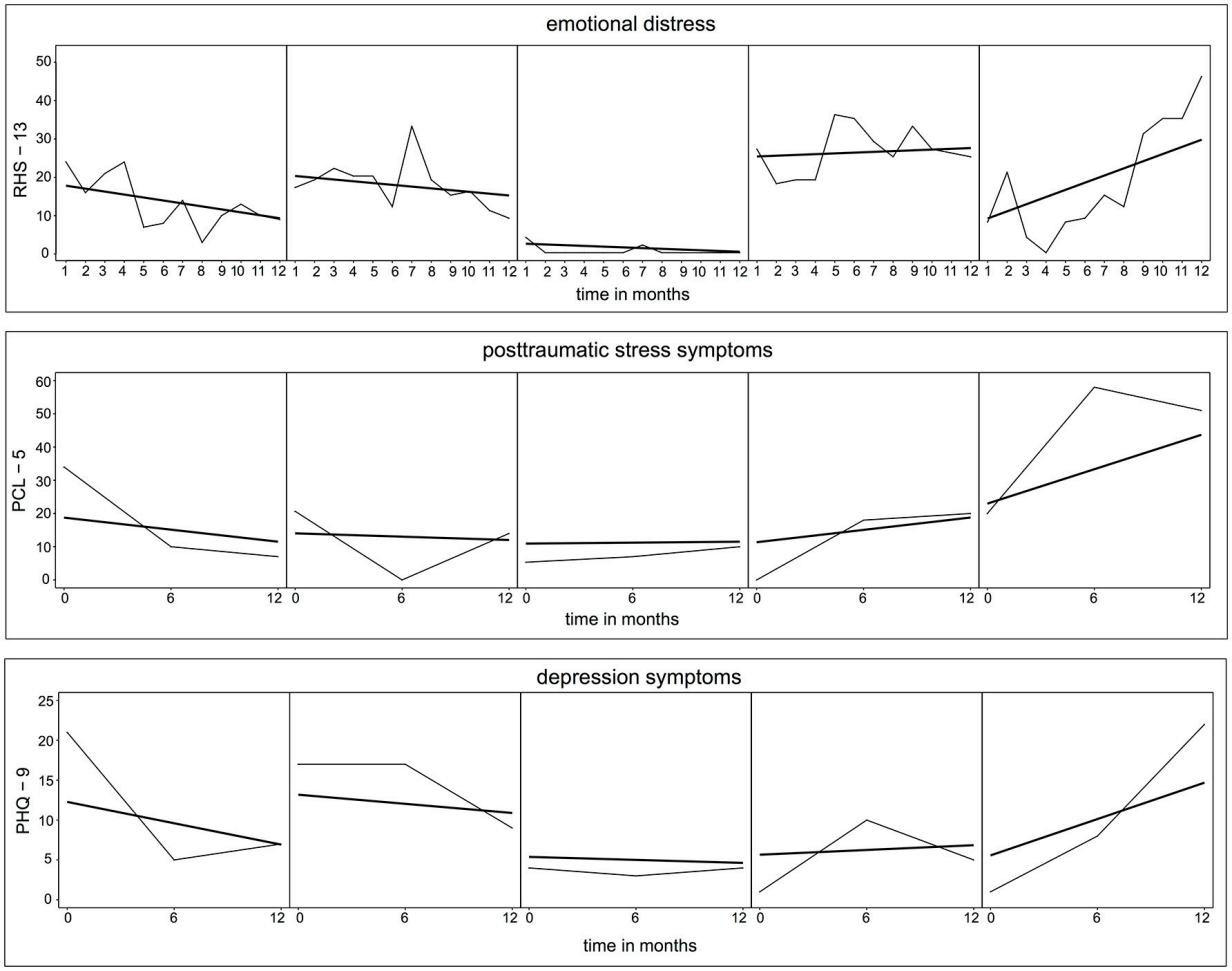
Raw and fitted individual time courses of emotional distress, posttraumatic stress symptoms, and depression symptoms for exemplary participants. Thin lines represent the actual course of symptoms. Bold lines depict the individually predicted LGMs. Selection procedure: Participants were ordered by the gradient of their slope and based on the quartiles exemplary participants were chosen.

First, we examined the course of symptoms for the response variables RHS-13, PCL-5, and PHQ-9 by including the fixed effect time only, and subject and time as a random slope. For all three symptom parameters, time did not represent a significant factor for the linear growth curve of the symptoms (RHS-13: estimate = −0.13, SE = 0.14, t = −0.95, *p* = 0.342; PCL-5: estimate = 1.22, SE = 1.36, *t* = 0.90, *p* = 0.373; PHQ-9: estimate = −0.11, SE = 0.63, *t* = −0.18, *p* = 0.856; for further information on the estimates see Supplementary Table 3). Respectively, in Figure 2, no change in the symptom level of emotional distress, PTSD, and depression could be detected for an average person of the sample. However, the fitted lines for the individuals show considerable between-subject variability of the intercepts and also of the slopes. The within-subject variability is illustrated in Figure 3 on exemplary raw and fitted courses of symptoms.

### Individual symptom changes

The majority of participants showed no significant change in PTSD and depression symptoms between t0 and t12. For PTSD symptoms, 24% (*n* = 9 of 38) of the participants showed a worsening and 13% (*n* = 5 of 38) an improvement of their symptoms. No clinically significant changes were found in 40% (*n* = 15 of 38) of the participants showing constantly subclinical and 24% (*n* = 9 of 38) showing ongoing clinically relevant PTSD symptoms. Results for changes in the depression symptoms are comparable, showing a worsening in 16% of the participants (*n* = 6 of 38), an improvement in 24% (*n* = 9 of 38), and 53% (*n* = 20 of 38) with subclinical depression symptoms, and 8% (*n* = 3 of 38) with ongoing clinically relevant depression symptoms.

### Predictors for the course of mental health symptoms

#### Emotional distress

For the RHS-13, predictors detecting changes within the year were examined and tested with LGMs (see Table 2 for the estimates). By including occupation (school, work, or apprenticeship) and time as fixed effects in the model, a significant main effect for occupation was found. That is, participants with a regular occupation reported less emotional distress.

**Table 2:** Parameter estimates for linear growth models—Refugee Health Screener-13.

**Models for RHS**	**Predictor occupation^a^**	**Predictor physician^a^**	**Predictor positive event^a^**	**Predictor negative event^a^**
**FIXED EFFECTS (INTERCEPT, SLOPES)**				
Intercept, estimate (SE), 95% CI	19.37 (1.77), [15.89, 22.85]	18.95 (1.57), [15.87, 22.03]	19.75 (1.57), [16.65, 22.84]	18.34 (1.56), [15.27, 21.42]
*t, p*	10.93, <0.001	12.09, <0.001	12.54, <0.001	11.72, <0.001
Predictor^a^, Estimate (SE), 95% CI	−4.51 (1.44), [−7.35, −1.68]	3.39 (0.93), [1.57, 5.21]	−1.32 (1.53), [−4.32, 1.68]	4.88 (1.14), [2.64, 7.12]
*t, p*	−3.13, 0.002	3.66, <0.001	−0.87, 0.386	4.28, <0.001
Time, estimate (SE), 95% CI	−0.17 (0.15), [−0.48, 0.13]	−0.12 (0.14), [−0.39, 0.16]	−0.13 (0.14), [−0.40, 0.14]	−0.09 (0.14), [−0.36, 0.18]
*t, p*	−1.14, 0.256	−0.85, 0.395	−0.94, 0.350	−0.66, 0.509
Interaction, estimate (SE), 95% CI	0.37 (0.20), [−0.02, 0.77]	−0.21 (0.15), [−0.50, 0.08]	−0.13 (0.26), [−0.64, 0.38]	−0.08 (0.19), [−0.46, 0.29]
*t, p*	1.86, 0.063	−1.43, 0.155	−0.50, 0.616	−0.43, 0.669
**RANDOM EFFECTS**				
Intercept, SD	10.60	11.02	11.09	10.84
Slope, SD	0.66	0.71	0.70	0.64
Intercept x time, correlation	−0.33	−0.26	−0.21	−0.17
Residuals, SD	5.66	5.93	6.00	5.88
Autocorrelation	0.26	0.18	0.19	0.24
**MODEL SELECTION**				
AIC random intercept	3494.60	3576.76	3680.20	3638.35
AIC random slope	3486.42	3566.78	3670.29	3632.75
LR χ^2^, *p*	12.19, 0.002	13.98, <0.001	13.91, 0.001	9.59, 0.008

*Corrected α = 0.013 with Benjamini-Hochberg procedure, SE = standard error; CI = confidence interval; AIC = Akaike information criterion; LR = likelihood ratio*;

a *predictor as defined in the respective column*.

In the second model, with the quantity of visits to physicians and time included as fixed effects, the quantity of visits revealed a significant main effect. Hence, participants who reported higher levels of emotional distress visited physicians more often.

Further, we constructed 2 models examining the effect of negative and positive events and time on the level of emotional distress. The most common positive events reported by the participants were the marriage of relatives or friends (*n* = 14), birth of relatives (*n* = 7), and celebrations (*n* = 4). The occurance of positive events was not found to be associated with emotional distress. However, including the occurance of negative events and time as fixed effects in the model, a significant main effect for negative events was found. Consequently, participants reporting more negative events showed a higher level of emotional distress. Negative events most often mentioned were the death of relatives (*n* = 26), severe disease of relatives (*n* = 18), and own illness (*n* = 16).

Sociodemographic variables (gender, education, age), traumatic events, and PMS did not reveal a significant main effect and / or interaction with time.

#### PTSD and depression

Sociodemographic variables as predictors for PTSD and depression were tested with LGMs. No significant main effects and interactions with time of the variables gender, education, age, partnership, duration of stay in Germany, duration of flight, asylum status at t0, and living situation at t0 were found. Furthermore, for depression, no significant effects with traumatic events or PMS were found (see Table 3).

**Table 3:** Parameter estimates for linear growth model—PCL-5 and PHQ-9.

	**PCL-5**		**PHQ-9**	
**Models for PCL-5 and PHQ-9**	**Predictor traumatic events^a^**	**Predictor PMLD^a^**	**Predictor traumatic events^a^**	**Predictor PMLD^a^**
**FIXED EFFECTS (INTERCEPT, SLOPES)**				
Intercept, estimate (SE), 95% CI	6.58 (3.80), [−0.99, 14.15]	21.36 (4.37), [12.65, 30.07]	7.29 (1.75), [3.80, 10.78]	8.61 (2.06), [4.49, 12.73]
*t, p*	1.73, 0.088	4.89, <0.001	4.16, <0.001	4.17, <0.001
Predictor^a^, Estimate (SE), 95% CI	1.89 (0.69), [0.51, 3.27]	−0.21 (0.13), [−0.48, 0.05]	0.25 (0.32), [−0.38, 0.89]	−0.03 (0.06), [−0.15, 0.10]
*t, p*	2.75, 0.008	−1.62, 0.113	0.80, 0.428	−0.47, 0.638
Time, estimate (SE), 95% CI	4.27 (3.08), [−1.86, 10.40]	−6.97 (2.77), [−12.49, −1.45]	−0.92 (1.46), [−3.81, 1.98]	−2.19 (1.34), [−4.86,0.47]
*t, p*	1.39, 0.170	−2.52, 0.014	−0.63, 0.531	−1.64, 0.105
Interaction, estimate (SE), 95% CI	−0.66 (0.54), [−1.75, 0.42]	0.26 (0.08), [0.10, 0.43]	0.12 (0.26), [−0.39, 0.63]	0.07 (0.04), [−0.01, 0.15]
*t, p*	−1.22, 0.226	3.18, 0.002	0.46, 0.646	1.72, 0.090
**RANDOM EFFECTS**				
Intercept, SD	0.32	0.36	0.13	0.18
Residuals, SD	13.61	12.13	6.31	5.75
Autocorrelation	0.37	0.35	0.31	0.35
**MODEL SELECTION**				
AIC random intercept	1088.33	855.24	888.92	698.58
AIC random slope	1090.97	858.79	892.73	702.07
LR χ^2^, *p*	1.36, 0.506	0.45, 0.800	0.20, 0.905	0.51, 0.775

*Corrected α = 0.025 with Benjamini-Hochberg procedure, SE = standard error; CI = confidence interval; AIC = Akaike information criterion; LR = likelihood ratio*;

a *predictor as defined in the respective column*.

When modeling the symptom level of PTSD along the time, the analysis revealed a significant fixed effect of the number of different traumatic event types, but no interaction with time (compare Table 3 for estimates). Consequently, across time, participants with a greater number of different types of traumatic events showed higher PTSD symptoms.

In a second model, PMS and time as fixed effects for the level of PTSD symptoms were tested. No significant fixed effect for PMS was found; however, a significant main effect for time and a significant interaction between PMS and time were found. The result revealed that participants who reported more PMS for the past year showed an increase in PTSD symptoms over the course of the year, while participants with less PMS showed a reduction in their symptoms (compare Table 3 for estimates).

To test whether both significant factors for PTSD—traumatic event types and PMS—remain significant in a combined model, we tested an exploratory LGM including traumatic event types, PMS, and time as main factors, and subject and time as random effects. Results revealed the same effects: a significant main effect was found for traumatic event types (estimate = 1.76, SE = 0.76, *t* = 2.31, *p* = 0.027), but not for PMS or time. A significant interaction was detected between PMS and time (estimate = 0.25, SE = 0.09, *t* = 2.89, *p* = 0.005), but not between traumatic events and time.

## Discussion

The aim of the present study was to depict the representational course of mental health symptoms in untreated refugees residing in Germany. Results indicate no overall change in emotional distress, PTSD, and depression symptoms over the course of 1 year. This can also be found on the individual level, with the majority showing no significant change in PTSD or depression symptoms. Risk factors for the severity and course of PTSD symptoms were the amount of different traumatic experiences and PMS. The intensity of emotional distress symptoms was associated with current negative life events, no occupation, and the amount of visits to physicians.

In line with previous longitudinal studies, the present findings showed on average no change in symptoms over time (22–24). However, other studies find a trend of improving or worsening symptoms of refugees' mental health over time (19, 21). At first sight, these results seem contradictory; however, we believe that the results mainly reflect the variability of mental health symptoms due to differing refugee characteristics, experiences in the home country, and the host country's surroundings—a conclusion also drawn for differing prevalence rates (12, 13). Accordingly, the results first and foremost show that the occurrence and the course of mental health symptoms in refugees depends on a variety of factors thereby also influencing the overall course of symptoms. This is also depicted in the high within- and between-subject variability found in the present study. Furthermore, around 40% show a significant worsening or improvement of their symptoms over time—this is in line with other studies finding a high heterogeneity in the courses of individuals (23, 24).

Consistent with previous research, we found high trauma exposure and PMS to be risk factors for PTSD symptoms (13, 32). Additionally, the exploratory LGM including both PMS and traumatic events revealed that traumatic events influence the severity of PTSD symptoms, while experiencing many PMS is associated with a deterioration of the PTSD symptoms over time. This finding highlights the importance of both traumatic events and PMS. To our knowledge, this result is new, as previous studies including PMS were mainly cross-sectional or focused on particular PMS (13, 30). However, despite of the longitudinal design, the causality of this relationship cannot be determined. While most cross-sectional literature assumes that PMS influence mental health (30), a longitudinal study by Tingvold et al. (31) showed that psychological distress leads to more acculturative hassles a decade later. Accordingly, an interaction between mental health and PMS seems to be likely. Furthermore, we looked in detail at particular stressors present in the host country. Thereby, we found a surprisingly high occurrence of negative events in the examined sample. The experience of those negative events was associated with higher levels of emotional distress at the according time point. This goes in line with other studies reporting that negative events in the host country or bad news about family members in the home country negatively influence mental health (61–63). The finding that a regular occupation such as work, school, or apprenticeship goes along with lower emotional distress is consistent with the literature (14, 64). However, the question of whether employment and mental health symptoms interact or lead to the respective other one remains open.

The heterogeneity of the naturalistic course of symptoms implies that especially vulnerable persons with a variety of risk factors need to be identified and suitable interventions should be offered. The influencing factors found in this study correspondingly lead to certain implications for practice and future research: Frequent visits to physicians can be an indicator of mental health problems. Together with other studies showing that refugees tend to receive little specialized treatment and are often not or wrongly diagnosed (65), this leads to the conclusion that physicians and mental health specialists should cooperate to provide the best possible and timely treatment - for example, by conducting mental health screenings in medical practices and referring those with mental health issues to the according mental health experts. Furthermore, appropriate treatment should be offered to reduce the effect of traumatic experiences on the mental health. Thereby, trauma-focused psychotherapeutic treatment shows good results in decreasing related mental health symptoms (66, 67).

The present study has certain limitations: The examined sample is a convenience sample, including 5 family dyads. Further, refugees receiving treatment—that is, especially those with more severe mental health symptoms—were excluded from this study. The slightly differing characteristics between study completers and dropouts with regard to the number of traumatic events and duration of stay in Germany might be due to the distinct characteristics of the participants being deported to their home countries during the study period. In addition, we cannot rule out that the use of the two somewhat differing PTSD instruments and traumatic event checklists in the baseline assessment due to the change from DSM-IV to DSM-5 led to a potential measuring inaccuracy. The assisted self-ratings and the interviews rely on the participants' subjective reports.

### Conclusion

On average, no change in the course of mental health symptoms could be detected—however, on an individual level, an improvement or worsening of symptoms was observed in around 40% of the cases. Traumatic experiences seem to lead to generally higher PTSD symptoms, while PMS experienced during the study period seem to be related to increasing PTSD symptoms over time. Emotional distress is associated with unemployment, current negative life events, and visits to physicians. Consequently, intensifying the cooperation of physicians and mental health experts to identify mental health problems in refugees is indicated. Further, evidence-based psychotherapeutic treatment is needed to reduce the mental suffering and allow integration into the host society, thus breaking the cycle of PMS and mental dysfunction.

## Ethics statement

This study was carried out in accordance with the recommendations of the Declaration of Helsinki and the Ethics Committee of the University of Konstanz, Germany. The protocol was approved by the Ethics Committee of the University of Konstanz, Germany. All subjects gave written informed consent in accordance with the Declaration of Helsinki and the Ethics Commitee of the University of Konstanz, Germany.

## Author contributions

EK, TE, KH, and MS developed the study concept and design. EK, KH, and MS collected data. EK guided the study realization, performed the data analyses and interpretation of findings, and wrote the first draft of the manuscript. IS and TE supervised the data analyses and interpretation. All authors revised the manuscript. All authors read and approved the final manuscript.

### Conflict of interest statement

The authors declare that the research was conducted in the absence of any commercial or financial relationships that could be construed as a potential conflict of interest.
