# Supplementary material for: Course of Mental Health in Refugees—A One Year Panel Survey
Source: Front Psychiatry. 2018 Aug 3;9:352. doi: 10.3389/fpsyt.2018.00352 (PMC6086111; doi:10.3389/fpsyt.2018.00352)
Supplement: Supplementary file 1 [file Table_1.PDF]

Supplementary 1. Comparison of completers and dropouts

Characteristic	Total sample (N = 57)	Completers (*n* = 44)	Dropouts (*n* =13)	Statistic	*p* value
Female sex, No. (%)	34 (60)	27 (61)	7 (54)	LR χ^2=.23	.750
Age, *M* (*SD*, range), years	30.3 (11.5, 12-56)	29.7 (11.6, 12-56)	32.5 (11.3, 14-56)	t(55)=-.76	.453
Education, *M* (*SD*, range), years	8.0 (4.1, 0-15)	7.7 (4.1, 0-13)	8.9 (4.1, 0-15)	t(55)=-.94	.353
Country of origin, No.				LR χ^2=15.63	.081
Syria	22	18	4		
Afghanistan	15	14	1		
Iran	7	5	2		
Iraq	4	3	1		
Macedonia	4	1	3		
Albania	1	0	1		
Kosovo	1	0	1		
Nigeria	1	1	0		
Russia	1	1	0		
Togo	1	1	0		
Stay in Germany, *M* (*SD*, range), months	9.3 (6.6, 2-36)	7.9 (5.3, 2-28)	13.9 (8.3, 6-36)	U=118.50, z=-3.20	.001**
Duration of flight, M (SD, range), months	10.6 (17.7, 0-75) [a]	11.2 (18.6, 0-75) [b]	8.4 (14.5, 0-38) [c]	t(53)=.48	.634
Core family members in Germany, No. (%)	41 (72)	32 (73)	9 (70)	LR χ^2=.41	.709
Accommodation, No. (%)				LR χ^2=7.04	.057
Emergency shelter	23 (40)	21 (48)	2 (15)		
Standard refugee accommodation	22 (39)	13 (30)	9 (69)		
Private accommodation	12 (21)	10 (23)	2 (15)		

Supplementary 1. Comparison of completers and dropouts (continued)

	Total sample (N = 57)	Completers (*n* = 44)	Dropouts (*n* =13)	Statistic	*p* value
Asylum status, No. (%)				LR χ^2=6.19	.068
First instance application	40 (70)	31 (71)	9 (69)		
Rejection	9 (16)	5 (11)	4 (31)		
Recognition	8 (14)	8 (18)	0 (0)		
Traumatic Events, *M* (*SD*, range)	4.9 (2.6, 1-12)	5.3 (2.5, 1-12)	3.4 (2.5, 1-10)	t(55)=2.46	.017*
PHQ sum score, *M* (*SD*, range)	8.2 (6.1, 0-27)	7.8 (6.2, 1-27)	9.4 (5.4, 0-19)	t(55)=-.81	.424
PTSD sum score, *M* (*SD*, range)	15.4 (12.6, 0-48)	14.9 (11.9, 0-48)	17.1 (14.9, 0-38)	t(55)=-.56	.578
Depression diagnosis, No. (%)	9 (16)	7 (16)	2 (15)	LR χ^2=.00	1.00
PTSD diagnosis, No. (%)	18 (32)	13 (30)	5 (39)	LR χ^2=.36	.735

Notes. LR χ^2= likelihood ratio χ^2, [a] n = 55, [b] *n* = 43, [c] *n* = 12.
