# Supplementary material for: Course of Mental Health in Refugees—A One Year Panel Survey
Source: Front Psychiatry. 2018 Aug 3;9:352. doi: 10.3389/fpsyt.2018.00352 (PMC6086111; doi:10.3389/fpsyt.2018.00352)
Supplement: Supplementary file 2 [file Table_2.pdf]

Supplementary 2. Self-constructed questions in the assisted self-report

1. Did you follow a (regular) occupation in the last month?	☐ no ☐ yes<br><br>If yes, what kind of occupation?<br>☐ work<br>☐ apprenticeship<br>☐ school<br>☐ language course<br>☐ other
2. Did your housing situation change in the last month?	☐ no ☐ yes<br><br>If yes, where are you living now?<br>☐ transfer to a refugee accommodation<br>☐ transfer to another refugee accommodation<br>☐ transfer within a refugee accommodation<br>☐ transfer to an apartment/ house<br>☐ transfer to a supervised housing group<br>☐ other change in housing situation
3. Has there been any new development regarding your application for asylum in the last month?	☐ no ☐ yes<br><br>If yes, what kind of change?<br>☐ rejection<br>☐ approval<br>☐ letter of unknown content<br>☐ other
4. Did you see a doctor or therapist in the last month?	☐ no ☐ yes<br><br>If yes, how many times did you see the according professionals?<br>☐ psychotherapist<br>☐ psychiatrist<br>☐ general practitioner<br>☐ other doctor<br>☐ inpatient health care
5. Have there been special life events in the last month (e.g. disease, death of relative, wedding etc.)?	☐ no ☐ yes<br><br>If yes, which kind of event?<br>☐ positive event, specify: ____________________<br>☐ negative event, specify: ____________________
