# Supplementary material for: Course of Mental Health in Refugees—A One Year Panel Survey
Source: Front Psychiatry. 2018 Aug 3;9:352. doi: 10.3389/fpsyt.2018.00352 (PMC6086111; doi:10.3389/fpsyt.2018.00352)
Supplement: Supplementary file 3 [file Table_3.PDF]

Supplementary 3. Parameter Estimates for linear growth models with fixed effect time – RHS-13, PCL-5, and PHQ-9

Models	RHS-13	PCL-5	PHQ-9
Fixed effects (intercept, slopes)			
Intercept, estimate (SE)	19.54 (1.58)	15.75 (1.76)	8.47 (.79)
t, p	12.38, < .001	8.93, < .001	10.73, < .001
Time, estimate (SE)	-.13 (.14)	1.22 (1.36)	-.11 (.63)
t, p	-.95, .342	.90, .373	-.18, .856
Random Effects			
Intercept, SD	11.18	.22	.10
Slope, SD	.72	2.89	1.30
Intercept x time, correlation	-.24	.18	-.05
Residuals, SD	6.02	13.57	6.11
Autocorrelation	.20	.39	.31

Notes. RHS-13 = Refugee Health Screener – 13, PCL-5 = Posttraumatic Stress Disorder Checklist-5, PHQ-9 = Patient Health Questionnaire – 9, SE = standard error.
